# Blocking EGFR with nimotuzumab: a novel strategy for COVID-19 treatment

**DOI:** 10.2217/imt-2022-0027

**Published:** 2022-03-21

**Authors:** Henrry Diaz Londres, Jorge Jiménez Armada, Aray Hernández Martínez, Anselmo A Abdo Cuza, Yamilka Hernández Sánchez, Aylin Granado Rodríguez, Sarahy Sepúlveda Figueroa, Egda M Llanez Gregorich, Mery L Torres Lahera, Francisco Gómez Peire, Teresita Montero González, Yaneth Zamora González, Ana L Añé Kouri, Addys González Palomo, Mayelin Troche Concepción, Loipa Medel Pérez, Patricia Lorenzo Luaces-Alvarez, Daymys Estévez Iglesias, Danay Saavedra Hernández, Mayra Ramos Suzarte, Tania Crombet Ramos

**Affiliations:** ^1^Julio Trigo Hospital, Havana, Cuba; ^2^Salvador Allende Hospital, Havana, Cuba; ^3^Medical & Surgical Research Center (CIMEQ), Havana, Cuba; ^4^Luis Díaz Soto Hospital, Havana, Cuba; ^5^National Institute of Hematology & Immunology, Havana, Cuba; ^6^Center of Molecular Immunology (CIM), Havana, Cuba

**Keywords:** COVID-19, EGFR, fibrosis, inflammation, monoclonal antibody, nimotuzumab, SARS-CoV-2

## Abstract

**Background:** Lung injury and STAT1 deficit induce EGFR overexpression in SARS-CoV-2 infection. **Patients & methods:** A phase I/II trial was done to evaluate the safety and preliminary effect of nimotuzumab, an anti-EGFR antibody, in COVID-19 patients. Patients received from one to three infusions together with other drugs included in the national guideline. **Results:** 41 patients (31 severe and 10 moderate) received nimotuzumab. The median age was 62 years and the main comorbidities were hypertension, diabetes and cardiovascular disease. The antibody was very safe and the 14-day recovery rate was 82.9%. Inflammatory markers decreased over time. Patients did not show signs of fibrosis. **Conclusion:** Nimotuzumab is a safe antibody that might reduce inflammation and prevent fibrosis in severe and moderate COVID-19 patients.

**Clinical Trial Registration:** RPCEC00000369 (rpcec.sld.cu).

## Plain language summary

**Background:** After SARS-CoV-2 infection, many cells in the lung express a new receptor called EGFR. Overexpression of EGFR can worsen the pulmonary disease and provoke fibrosis. **Patients & methods:** The initial impact of using a drug that blocks EGFR, nimotuzumab, was evaluated in COVID-19 patients. **Results:** 41 patients received nimotuzumab by the intravenous route together with other medications. The median age was 62 years, and patients had many chronic conditions including hypertension, diabetes and cardiac problems. Treatment was well tolerated and 82.9% of the patients were discharged by day 14. Serial laboratory tests, x-rays and CT scan evaluations showed the improvement of the patients. **Conclusion:** Nimotuzumab is a safe drug that can be useful to treat COVID-19 patients.

COVID-19 is a gradually evolving pathology characterized by different molecular mechanisms [1]. The first analyses of COVID-19 patients in China suggested that the virus might not be solely responsible for lung damage, but that a hyper-reactive immune response could contribute to the pathogenesis of the disease [2].

Several SARS-CoV-2 genes induce a deficit of STAT1, followed by a hyperactivation of STAT3 [3]. The STAT1 loss, together with the lung injury, induces *EGFR* overexpression in the infected alveolar epithelial cells. Activation of EGFR leads to additional activation of STAT3. Furthermore, in cells infected with SARS-CoV-2, a positive response circle is established between STAT3 and PAI-1 [3]. Upregulation of PAI-1 leads to a coagulopathy characterized by intravascular thrombi [3].

EGFR is a membrane glycoprotein with tyrosine kinase activity whose physiological role is to regulate epithelial tissue development and homeostasis [4]. There is significant evidence of EGFR’s role in fibrosis. According to Venkataran *et al.*, EGFR signaling remains active after the clearance of SARS-CoV and leads to fibrosis [5]. EGFR is also upregulated during the pathological remodeling of the lung in patients with advanced cystic fibrosis [6], while Ishii *et al.* found that gefitinib, an EGFR tyrosine kinase inhibitor, had a protective effect on the lung fibrosis induced by bleomycin [7].

*EGFR* is also overexpressed in squamous carcinomas or adenocarcinomas of different localization [4]. Knowledge of the role of the EGFR in malignant tumors has advanced enormously over the last 20 years, and several therapeutic drugs such as tyrosine kinase inhibitors and anti-EGFR monoclonal antibodies have been registered for epithelial-derived tumors [8–10]. Nimotuzumab is a humanized antibody that targets EGFR [11]. The antibody prevents receptor dimerization, thus inhibiting tyrosine kinase activity and interfering with the cell signaling pathways involved in proliferation, inflammation, angiogenesis and survival [12,13]. Nimotuzumab decreases IL-6 in mice xenografted with pancreatic tumor cells [13]. Blocking EGFR signaling is not the only mechanism of action underlying the efficacy of nimotuzumab. *In vitro* studies demonstrated that nimotuzumab exerts its effects partially through antibody-dependent cell-mediated cytotoxicity and by activating dendritic cells and specific CD8^+^ T cells [14,15].

Nimotuzumab efficacy results have been reported in more than 40 clinical trials, conducted primarily in patients with advanced cancer, including glioblastoma, squamous cell carcinoma of the head and neck, and esophageal, nasopharyngeal, pancreatic and non-small-cell lung cancers [14,16–21].

Given that acute lung injury and functional loss of STAT1 can lead to *EGFR* overexpression, we hypothesized that EGFR blockade with nimotuzumab would be an effective strategy to control inflammation and to prevent an excessive fibrosis after SARS-CoV-2 infection.

## Patients & methods

EGFR expression was evaluated by immunohistochemistry in lung samples from COVID-19 deceased patients. 20 lung fragments were obtained from the Department of Pathology of the hospitals Luis Díaz Soto and Salvador Allende (Havana, Cuba). Fragments were fixed in formaldehyde and embedded in paraffin blocks. Slides with lung tissue sections were deparaffinized and washed in phosphate buffer solution and distilled water. The endogenous peroxidase activity (Dako, CA, USA) was blocked and EGFR antigenic resuscitation was done using proteinase K (Dako). Samples were incubated with a murine anti-human EGFR monoclonal antibody for EGFR3 for 1 h. Subsequently, the sections were incubated with horseradish peroxidase (Dako) and the enzymatic reaction was developed with diaminobenzidine tetrahydrochloride (Dako). Tissue sections were also contrasted with Mayer’s hematoxylin (Dako). Positive expression was defined by the presence of a brown color located in the plasma membrane of the different cell subtypes. The percentage of positive cells per histological field was quantified on five images digitized with a DP20 digital camera coupled to an optical microscope (Olympus CX31, Tokyo, Japan), at 40 × and 100 × magnification.

A prospective, noncontrolled, multicenter phase I/II trial was done to assess the effect and tolerability of nimotuzumab in individuals with COVID-19. Diagnosis was confirmed by PCR. Nimotuzumab was administered together with the standard of care (SOC): low-molecular-weight heparin, steroids and antibiotics, according to the national COVID-19 guideline [22]. Patients could also receive CIGB-258, a peptide with immunoregulatory properties which obtained Emergency Use Authorization from the Cuban regulatory agency [23]. Inclusion criteria were the following: express willingness of the patient, any gender, any ethnicity and age ≥18 years. In addition, patients should have severe disease according to one of the following conditions: oxygen saturation (SpO_2_) <94% on room air at sea level or need for oxygen therapy to maintain SpO_2_ >93%, a pressure of arterial oxygen to fractional inspired oxygen (PaO_2_/FiO_2_) <300 mm Hg, a respiratory rate >30 breaths/min or lung infiltrates >50%. Patients with moderate symptoms at high risk of worsening were also included. Subjects with moderate illness were those with evidence of lower respiratory disease according to the clinical assessment or imaging, who had an SpO_2_ ≥94% on room air at sea level. High-risk patients were those aged ≥65 years or with comorbidities such as diabetes mellitus, hypertension, chronic kidney disease, cardiovascular disease, immunodeficiency, obesity or cancer. The protocol was approved by the ethics committees of the participating hospitals and by the National Regulatory Agency, CECMED. All patients signed the informed consent. The protocol was listed in the public registry of clinical trials (https://rpcec.sld.cu/ensayos/RPCEC00000369-En).

Treatment consisted of nimotuzumab intravenous infusions up to a maximum of three doses, administered every 72 h. The recommended number of doses was two for moderate patients and three for individuals with severe disease. The main criterion for discontinuation was the occurrence of serious adverse events related to the investigational product. The loading dose was 200 mg, while subsequent doses of the antibody consisted of 100 mg. Nimotuzumab was diluted in 250 ml of saline solution (0.9%) and administered over 2 h.

The objectives of the study were to evaluate the preliminary safety and effect of nimotuzumab in the treatment of severe or moderate COVID-19. Adverse events were classified according to frequency, intensity, causal relationship and consequences for the subject. Intensity was graded according to the Common Terminology Criteria for Adverse Events, version 5 [24].

The main effect variables were the following: rate of patients recovered 14 days after the first infusion, length of stay in the intensive care unit (ICU), rate of patients whose PO_2_/FiO_2_ ratio improved and rate of patients requiring mechanical ventilation after nimotuzumab. Hematology including neutrophil, lymphocyte and platelet counts was evaluated before nimotuzumab and every 48 h. Other biochemical parameters including creatinine, aspartate aminotransferase, alanine aminotransferase, C-reactive protein, ferritin, lactate dehydrogenase (LDH) and D-dimer were measured over time. In addition, IL-6 and PAI-1 were measured using an IL-6 Quantikine^®^ ELISA Kit (Cat# S6050; R&D Systems, MN, USA) and a quantitative PAI-1 ELISA set (BE59351; BL International GmbH, Hamburg, Germany). PAI-1 was measured in plasma. The evolution of the lung lesions was measured by serial x-ray or CT scan.

Demographics, comorbidities and previous and concurrent therapies were reported in the two disease severity groups. Descriptive statistics were used to display qualitative variables, while mean and standard deviation or median and interquartile range were used for quantitative variables with normal or non-normal distribution, respectively. Receiver operating characteristic curves were built for all baseline laboratory variables to predict COVID-19 mortality. The odds ratios (ORs) for the variables associated with the highest lethality risk were determined. Data processing was done with the R package (www.r-project.org/) and SPSS-25 software (IBM Corp., NY, USA).

## Results

**Figure 1.**
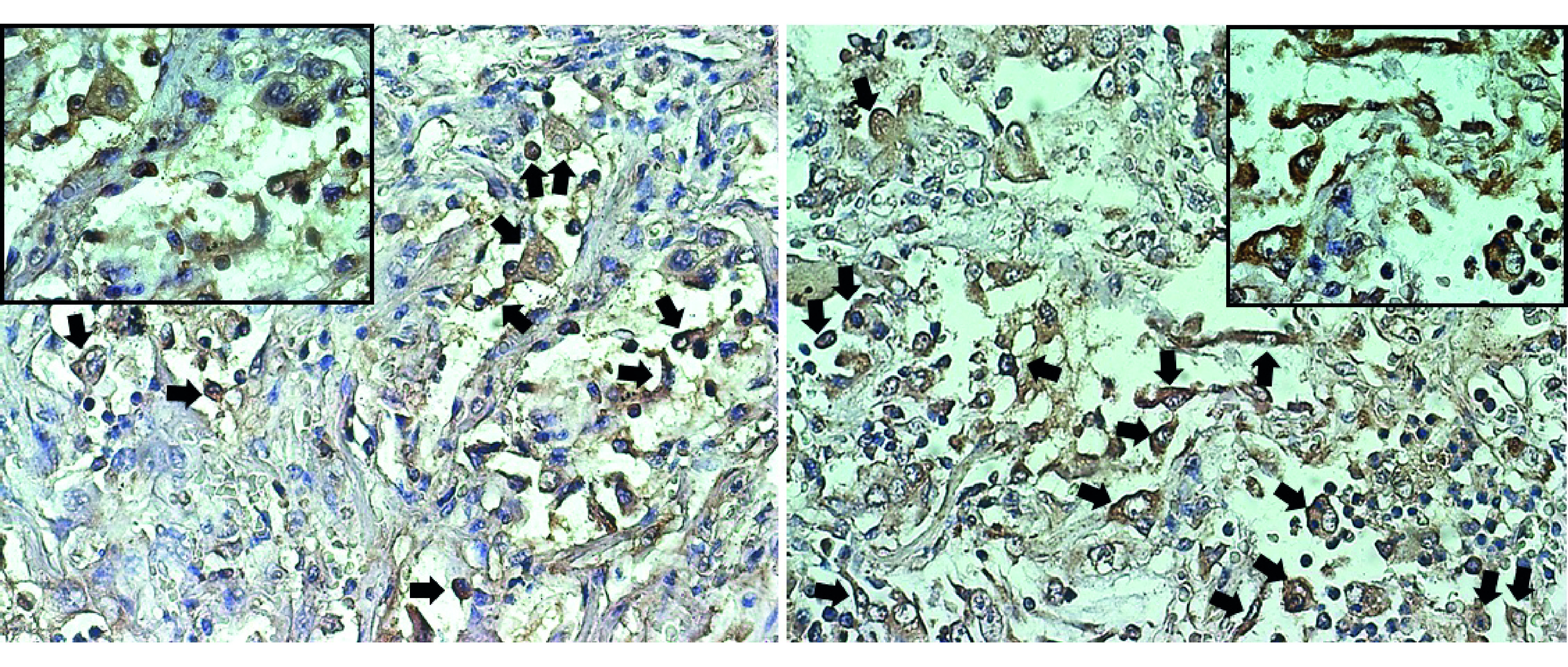
Representative microscopic images (40× and 100×) of the EGFR expression in the lung tissue from two deceased COVID-19 patients. Black arrows show the positive cells including pneumocytes, alveolar macrophages and fibroblasts.

EGFR expression was detected in the lung necropsy specimens of the 20 evaluated patients who died from COVID-19. In 19 samples (95%), more than 60% of the cells showed EGFR expression, while one tissue section had 25% EGFR positivity. Positive cells morphologically resemble type I and II pneumocytes, alveolar macrophages and fibroblasts. Figure 1 shows the EGFR expression in the lung sections from two representative deceased COVID-19 patients.

After the demonstration of EGFR expression in the lung tissue from deceased COVID-19 patients, a clinical trial using the anti-EGFR monoclonal antibody nimotuzumab was performed. From 19 May to 19 June 2021, 41 patients (31 severe and 10 moderate) were included from three hospitals of Havana. Overall, 23 male and 18 female patients were included (Table 1). The median age was 62 years (range: 32–83) and the main comorbidities were hypertension, diabetes mellitus, cardiovascular disease and obesity (Table 1). Most individuals received nimotuzumab concomitantly with the standard therapy including low-molecular-weight heparin, steroids and antibiotics (Table 2). A small group of patients (13; 31.7%) also received CIGB-258 concomitantly with nimotuzumab (Table 2) [23].

**Table 1.** Demographics and comorbidities of patients at baseline.

Demographic	Severe		Moderate		Total	
	n	%	n	%	n	%
Total population	31	100	10	100	41	100
Gender						
– Female	13	41.9	5	58.1	18	43.9
– Male	18	58.1	5	50	23	56.1
Skin color						
– White	3	30	17	54.8	20	48.8
– Mixed	4	40	8	25.8	12	29.3
– Black	2	20	3	9.7	5	12.2
– ND	1	10	3	9.7	4	9.8
Patients with at least one comorbidity	27	87.1	7	70	34	82.9
Patients with two or more comorbidities	15	48.4	5	50	20	48.8
Hypertension	19	61.3	6	60	25	61
Cardiovascular diseases	5	16.1	1	10	6	14.6
Diabetes mellitus	11	35.5	5	50	16	39
Bronchial asthma	4	12.9	0	0	4	9.7
Chronic obstructive pulmonary disease	1	3.2	0	0	1	2.4
Obesity	5	16.1	1	10	6	14.6
Stroke	1	3.2	0	0	1	2.4
Hyperthyroidism	0	0	2	20	2	4.8
Hypothyroidism	0	0	1	10	1	2.4
Cancer	1	3. 2	0	0	1	2.4
Pericarditis	2	6.4	0	0	2	4.8
Rheumatoid arthritis	0	0	1	10	1	2.4
Age (years)						
– Mean ± SD	59.8 ± 15		59.5 ± 12		59.7 ± 14	
– Median ± IR	62 ± 26		61 ± 18		62 ± 22	
– Min–max	32–83		35–74		32–83	

IR: Interquartile range; ND: No data; SD: Standard deviation.

**Table 2.** Concurrent therapies individuals received during nimotuzumab treatment.

Concurrent therapy	Severe		Moderate		Total	
	n	%	n	%	n	%
Recombinant IFN-α	0	0	3	30	3	7.3
Low-molecular-weight heparin	30	96.8	4	40	34	82.9
Steroids	28	90.3	3	30	31	75.6
Antibiotics	30	96.8	10	100	40	97.6
CIGB-258	12	38.7	1	10	13	31.7

All patients who entered the trial with a severe condition were treated at the ICU, while moderate patients received nimotuzumab at the hospital conventional ward. The time lag between the onset of symptoms and nimotuzumab treatment was 8.5 days, while for the severe patients the time interval between ICU admission and nimotuzumab was 3 days.

Seven patients (17.07%) received a single dose of nimotuzumab, 29 (70.7%) received two infusions, and five subjects (12.19%) required three doses of the antibody. Nimotuzumab was very safe. There were only four related adverse events in two patients (4.87%). The two individuals with adverse reactions both had severe disease; one subject developed grade 1 tremors, while the second patient presented grade 2 chills, headache and tremors. The events were classified as possibly related to nimotuzumab and occurred after the first antibody infusion. No grade 3 or 4 related adverse events were detected.

Eight patients (one moderate and seven severe; 19.5%) of the 41 receiving nimotuzumab required invasive mechanical ventilation. The mean time between nimotuzumab and the use of invasive mechanical ventilation was 3.6 days. 34 patients out of 41 (82.92%) recovered 14 days after receiving the first monoclonal antibody infusion. Regarding disease severity, nine of ten moderate patients (90%) recuperated, while 25 of the 31 severe patients (80.64%) were discharged by day 14. The median time at the ICU of the severely ill subjects was 9 days.

Surprisingly, patients who received nimotuzumab concomitantly with CIGB-258 and the SOC had a worse outcome as compared with patients who received nimotuzumab plus the SOC alone. Overall, eight of 12 severe patients (66.7%) who received CIGB-258 recovered, compared with 17 of 19 individuals (89.5%) treated with nimotuzumab plus steroids, anticoagulant and antibiotics. In the moderate setting, only one subject received nimotuzumab and CIGB-258, and did not recover. The rest of the moderate patients (nine) who received nimotuzumab plus the SOC rapidly improved. The most prevalent cause of death of the individuals receiving nimotuzumab and CIGB-258 was septic shock.

**Figure 2.**
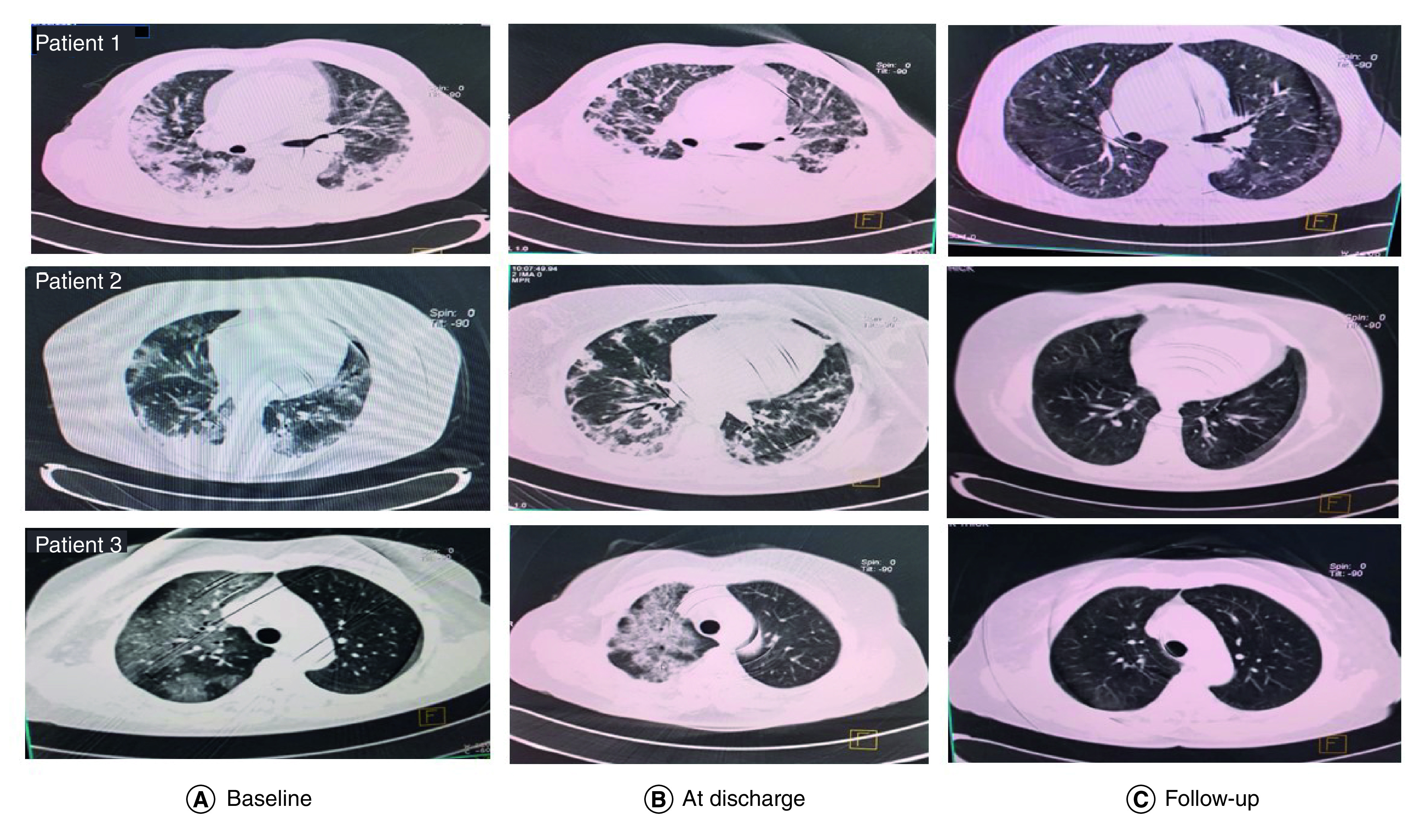
Axial chest CT scans of three patients treated with nimotuzumab. Sequential images on admission (column A), at discharge (column B) and follow-up (30–60 days after discharge). **(A & B)** Extensive areas of ground-glass opacities, airspace consolidation in exudative phase and decreased lung volumes in organizing and fibrotic phases. **(C)** Follow-up: the three patients showed resolution of the lung inflammatory lesions and no sign of fibrosis.

On day 7 of treatment, 76.2% of the subjects with a severe condition had an improved PO_2_/FiO_2_ ratio. The percentage of the affected area of both lung fields was calculated both before treatment with nimotuzumab and at the time of discharge. There was a significant reduction in the affected area of both lungs at discharge, according to the Wilcoxon matched-pairs signed rank test. CT scan images were acquired before and at discharge in 24 patients. At discharge, all patients but four (82.4%) had CT scan abnormalities including ground-glass opacities, consolidation, reticulation (crazy-paving appearance), septal thickening and decreased lung volumes. Follow-up CT scans were done in 15 patients after 30–60 days. Minor CT abnormalities, but no sign of fibrosis, persisted in one case (6%). Figure 2 shows serial images of three patients before treatment with nimotuzumab, at the time of discharge and 30–60 days after hospitalization.

Inflammatory markers including C-reactive protein, ferritin, LDH, neutrophil-to-lymphocyte ratio and D-dimer decreased over time. Receiver operating characteristic curves were performed to establish the correlation between laboratory parameters at baseline and the probability of dying of patients with severe disease. Patients with C-reactive protein values above 118.54 mg/l, LDH above 333 U/l, absolute leukocyte count above 8.95 × 10^9^/l and ferritin above 490.5 μg/l were more likely to die. Remarkably, the biomarkers associated with the highest lethality risk were LDH ≥333 U/l (OR: 11.25; 95% CI: 1.15–110.5) and absolute leukocyte count ≥8.95 × 10^9^/l (OR: 9.33; 95% CI: 0.96–90.9).

Serum IL-6 concentration was evaluated in 28 patients, 21 classified as severe and seven as moderate. The median IL-6 concentration at the time of inclusion in the clinical trial was 43.83 pg/ml for severe patients and 46.47 pg/ml for subjects with moderate disease. No significant differences were identified when comparing the two illness subsets (Mann–Whitney test: p > 0.05). At day 7, IL-6 increased in four patients of the 29 (13.7%), while it decreased or stabilized in the rest. Globally, the median IL-6 concentration diminished from 46.5 to 14.51 pg/ml at day 7. PAI-1 levels in plasma were evaluated only in five patients with moderate disease, on account of the limitations of the quantification system. Notably, PAI-1 was elevated in these five patients (reference values 4–43 ng/ml). The median PAI-1 level at baseline was 123 ng/ml and declined to 77.3 ng/ml at day 7 of treatment.

## Discussion

The symptoms of COVID-19 are extremely variable, ranging from minimal to significant hypoxia with acute respiratory distress syndrome (ARDS) and multi-organ failure, which can be fatal [1].

This clinical trial evaluated for the first time the effect of using an anti-EGFR inhibitor in the COVID-19 scenario. The overexpression of EGFR in the lung tissue from SARS-CoV-2 deceased patients was demonstrated. This finding further validates the approach of blocking EGFR as a tool to reduce inflammation or hypofibrinolysis and to prevent or revert fibrosis. Apart from in non-small-cell lung cancer, EGFR is not expressed in normal lung tissue [25–29].

According to the most recent publications, COVID-19 pathogenesis can be divided into three overlapping steps: pulmonary, proinflammatory and prothrombotic. In the pulmonary phase, the virus affects the epithelial alveolar cells, causing interstitial pneumonia. In the proinflammatory stage, infected target cells and lymphocytes further overproduce inflammatory cytokines that result in acute lung injury. The last stage is characterized by an uncontrolled coagulopathy [30]. Gautret *et al.* proposed a clinical spectrum including an acute virologic stage followed by a cytokine storm, a procoagulation disorder and an ARDS [31]; Lippi and coworkers divided the disease into at least five phases (incubation, respiratory, proinflammatory, prothrombotic, death or remission) [32].

We postulate that nimotuzumab can be used when there is evidence of lower respiratory disease, corresponding to the pulmonary or respiratory stage of the disease. At this stage, EGFR is overexpressed by the respiratory cells after the STAT1 loss and the acute lung damage [3]. The antibody can also be useful in the inflammatory and prothrombotic phases of the disease, given the crucial regulatory role of EGFR in inflammation and immunothrombosis. EGFR activation has been found to trigger the proinflammatory response and PAI-1 secretion [33,34]. Nimotuzumab was very safe, and only two patients had tremors, chills or headache. Adverse reactions commonly seen in cancer trials consist mostly of fatigue, nausea, vomiting, chills, anorexia and fever [16,20,35]. Other recently recommended anti-inflammatory drugs for COVID-19, like tocilizumab or baricitinib, might provoke serious adverse reactions including bacteremia and lung abscess [36] or grade 3 or 4 adverse events such as hyperglycemia, anemia, decreased lymphocyte count and acute kidney injury, respectively [37].

The clinical trial provided initial evidence that nimotuzumab, in combination with other drugs including steroids, decreases inflammatory markers, including IL-6. IL-6 is a pleiotropic cytokine that has a central role in the immune response as well as inflammation [38]. Remarkably, in our series, no differences were found between moderate and severe patients regarding IL-6 concentration. IL-6 levels did not further increase in 86% of the patients after blocking EGFR and treatment with steroids. Other molecules targeting the IL-6 receptor pathway, including tocilizumab or baricitinib, had significant clinical effect when combined with steroids [36,39].

Preliminary information on PAI-1 levels was also obtained. PAI-1 was elevated in the five evaluated patients. Previously, PAI-1 has been shown to be augmented in ARDS and particularly in COVID-19 [40], but notably, Cugno *et al.* did not find differences in PAI-1 concentration among patients with mild, moderate or severe disease [41]. As well as endothelial cells, senescent alveolar type II cells secrete PAI-1, which promotes a profibrotic phenotype [42]. According to Alberti *et al.*, EGFR activation triggers coexpression of IL-6 and PAI-1, via transcriptional activation of NF-κB [33]. The activation of the IL-6 pathway might also be the driver of the PAI-1 secretion during the cytokine storm [43]. Our initial findings on the reduction of PAI-1 could be attributed either to the direct inhibition of EGFR or to the IL-6 decrease.

In our dataset, 19.5% of the 41 patients needed mechanical ventilation after nimotuzumab. The recovery rates were 80.6 and 90% for severe and high-risk moderate patients, respectively. Even though a direct comparison is not possible, these figures compare favorably with other anti-inflammatory drugs that obtained emergency use authorization for COVID-19. The RECOVERY study evaluating the anti-IL-6R monoclonal antibody tocilizumab versus usual care, enrolled 4116 patients, mostly receiving noninvasive respiratory support or oxygen. Almost all patients received corticosteroids; recovery rates were 62 versus 58% in patients requiring noninvasive ventilation and 81 versus 77% in patients requiring solely oxygen [36]. No significant effect on subsequent ventilation was seen, and globally, 31% of the tocilizumab patients progressed to respiratory support [36]. In another double-blind study in 438 subjects with bilateral pulmonary infiltrates and hypoxemia (COVACTA), the mortality rate was 19.7 versus 19.4% for tocilizumab or placebo [44]. The incidence of mechanical ventilation among patients not ventilated at baseline was 27.9% (tocilizumab) versus 36.7% (placebo) [44]. Recently, a JAK inhibitor, baricitinib, showed clinical benefit in hospitalized patients. A randomized study of 1033 patients (67% moderate and 33% severe) compared remdesivir plus baricitinib versus remdesivir plus placebo [37]. The incidence of death or ventilation was lower in the experimental than in the control group (22.5 vs 28%) [37]. The second trial, by Marconi *et al.*, compared baricitinib plus SOC versus SOC in 1525 patients. Overall, 75% of the patients did not require ventilation or high-flow oxygen at baseline. The percentage of individuals who died or required ventilation or high-flow oxygen was 27.5% in the baricitinib cohort versus 30.5% in the control group [39].

After this phase I/II study was concluded, nimotuzumab was included in the national guideline to treat COVID-19 patients. In order to assess the impact of using nimotuzumab in the real-world scenario, the recovery rate was compared with a paired retrospective cohort. Control patients received standard treatment according to the national protocol, but not nimotuzumab. Overall, 1151 severe or critical patients receiving nimotuzumab and 969 matching controls were compared. The 14-day recovery rate of the nimotuzumab cohort was 79.8%, compared with 45.3% in the control group.

Treatment with CIGB-258, an immunoregulatory peptide, was not an exclusion criterion, given that this was the first trial designed to assess the impact of nimotuzumab in COVID-19-associated inflammation. Remarkably, combining two drugs like nimotuzumab or CIGB-258, which decrease IL-6 by different mechanisms, did not have an additive effect, and patients died mainly as a result of coinfections and septic shock. It is worth considering that infections are very frequent in hospitalized older patients bearing chronic diseases [45]. The use of IL-6 or IL-6R-blocking antibodies in combination with JAK inhibitors to treat COVID-19 patients is also not recommended.

Finally, our study found a significant reduction of the affected lung areas and no signs of fibrosis in those subjects evaluated 30–60 days after discharge. A recent meta-analysis in 250,351 survivors demonstrated that 65% of the patients had increased oxygen requirement while chest imaging abnormalities persisted in 62.2% of all survivors up to 6 months after recovery [46]. In our small series, CT scan anomalies persisted in only 6% of the patients evaluated up to 60 days after discharge. Long-term evaluation of the sequelae of the nimotuzumab-treated patients, including pulmonary function tests, is planned.

## Conclusion

In summary, our preliminary results suggest that nimotuzumab is a safe antibody that might reduce IL-6 and PAI-1 and prevent fibrosis in severe and moderate COVID-19 patients at high risk of aggravation. In spite of the patients’ poor prognosis, the ventilation rate was lower than 20% and the recovery rate was above 80% for the severe cases. These results should be interpreted with caution, given the small sample size and the uncontrolled nature of the trial. A larger series of patients has been evaluated, and a controlled trial in the COVID-19 or post-COVID setting is planned.

**Summary points**

- Lung injury and STAT1 deficit can induce EGFR overexpression in SARS-CoV-2-infected cells.
- EGFR overexpression further worsens inflammation, immune thrombosis and fibrosis.
- This clinical trial evaluated for the first time the effect of using an anti-EGFR antagonist (nimotuzumab) in combination with other drugs in the COVID-19 scenario.
- EGFR overexpression in the lung tissue from SARS-CoV-2 deceased patients was demonstrated.
- The antibody was very safe. There were only four related adverse events in two subjects.
- The 14-day recovery rate was 82.9%. Regarding disease severity, 90% of the moderate patients and 80.64% of the severe patients recuperated by day 14.
- Inflammatory markers decreased over time; IL-6 concentration diminished from 46.5 pg/ml to 14.51 pg/ml at day 7.
- None of the evaluated patients showed signs of fibrosis in the follow-up evaluation.
